# Using needle orientation sensing as surrogate signal for respiratory motion estimation in percutaneous interventions

**DOI:** 10.1007/s11548-017-1644-z

**Published:** 2017-08-01

**Authors:** Momen Abayazid, Takahisa Kato, Stuart G. Silverman, Nobuhiko Hata

**Affiliations:** 1 Department of Radiology, Brigham and Womens Hospital and Harvard Medical School, Boston, MA USA; 2 MIRA-Institute for Biomedical Technology and Technical Medicine (Robotics and Mechatronics), University of Twente, Enschede, The Netherlands; 3 Healthcare Optics Research Laboratory, Canon U.S.A., Inc., Cambridge, MA USA

**Keywords:** Machine learning, Motion compensation, Respiratory motion, Magnetic resonance imaging, Percutaneous needle insertion, Interventional radiology

## Abstract

**Purpose:**

To develop and evaluate an approach to estimate the respiratory-induced motion of lesions in the chest and abdomen.

**Materials and methods:**

The proposed approach uses the motion of an initial reference needle inserted into a moving organ to estimate the lesion (target) displacement that is caused by respiration. The needles position is measured using an inertial measurement unit (IMU) sensor externally attached to the hub of an initially placed reference needle. Data obtained from the IMU sensor and the target motion are used to train a learning-based approach to estimate the position of the moving target. An experimental platform was designed to mimic respiratory motion of the liver. Liver motion profiles of human subjects provided inputs to the experimental platform. Variables including the insertion angle, target depth, target motion velocity and target proximity to the reference needle were evaluated by measuring the error of the estimated target position and processing time.

**Results:**

The mean error of estimation of the target position ranged between 0.86 and 1.29 mm. The processing maximum training and testing time was 5 ms which is suitable for real-time target motion estimation using the needle position sensor.

**Conclusion:**

The external motion of an initially placed reference needle inserted into a moving organ can be used as a surrogate, measurable and accessible signal to estimate in real-time the position of a moving target caused by respiration; this technique could then be used to guide the placement of subsequently inserted needles directly into the target.

## Introduction

Percutaneous image-guided interventional radiology procedures in the chest and upper abdomen are commonly used for a variety of procedures such as biopsy, fluid collection drainages and tumor ablation [2, 24]. Both ultrasound and computed tomography (CT) are typically the imaging guidance modalities of choice procedures [1]. Magnetic resonance imaging (MRI) and PET/CT may also be used. Whichever imaging modalities are used for interventions; accurate targeting of the lesions is critical for successful completion of the interventions [3]. In contrast, inaccurate targeting can cause misdiagnoses in a case of biopsy, and insufficient treatment and recurrences in case of ablation therapies. The most common cause of inaccurate placements of interventional instruments is respiratory motion [5, 11]. Interventional radiologists have proposed approaches to mitigate the error caused by respiration motion by either an active or passive approach [19, 39]. The active approach is breath holding during imaging and instrument insertions. Ideally, the breath should be held consistently (at the same respiratory phase) each time during imaging or needle manipulation, so that the target maintains the same, predictable position throughout the interventional procedure [19]. A study by Zhou et al. [39] reported, however, that 10–15% of the patients cannot hold their breath for a sufficient time; this often leads to inaccurate placements. Breath-holding aids have been used; for example, a bellows device leads to an LED display that shows the patient the level of the breath that can improve consistency [8]. Passive approaches compensate for respiratory motion without breath holds. These approaches require the detection of the targeted lesions location in real-time during respiration and then adjust the needle insertion angle and position during interventional procedures to compensate for the respiratory-induced motion. However, detecting the position of the lesion is challenging in real-time [16]. Currently, there are four main methods of tracking a lesion and consequently correcting for its motion during an image-guided procedure: (1) imaging of the lesion [19]; (2) imaging of fiducial markers implanted in or near the lesion in case the lesion is not easily visible [4, 32]; (3) detection of an active or passive signaling device implanted in or near the lesion [25, 27, 31]; and (4) inference of the lesion position/motion from a respiratory surrogate signal [26]. The advantage of using surrogate signals is that they can provide motion data with higher refresh rate than the imaging modality that can detect small lesions in liver which is MRI. Other imaging modalities do not provide images with sufficient contrast to detect small lesions in liver. Additionally, the approach of implanting fiducial markers or signaling devices is adding a new challenging task because the implant needs to be placed accurately in close vicinity to the lesion.

The main requirements for surrogate signals are that they should have a strong relationship with the actual target motion, and that they can be acquired with sufficiently high temporal frequency to estimate the motion in real-time [25]. An example of a respiratory surrogate signal is respiratory bellows measurements. Respiratory bellows are used as an alternative means of measuring respiratory position during MR imaging [30]. It consists of an air filled bag, which is wedged between the subjects’ abdomen or chest and a firm surface such as an elasticated belt. The motion of the abdomen or chest during respiration causes air to be expelled from the bellows and a sensor measures the flow of the air. The bellows are not widely used due to technical issues regarding lack of information about breathing amplitude. Spirometer measurements have also been proposed for use as a surrogate signal for respiratory motion models [15, 21, 22, 38]. The spirometer measures the air flow to and from the lungs and is commonly used for testing pulmonary function [23]. Some authors have proposed using the motion of the diaphragm surface as surrogate signal [17]. The diaphragm surface could be measured using fluoroscopy or ultrasound. A common means of acquiring respiratory surrogate data for a range of motion modeling applications has been to track the motion of one or more points on the skin surface of the chest or abdomen using optical tracking, electromagnetic tracking or laser-based tracking systems [14, 15, 23]. However, the effectiveness of using skin surface motion to estimate the organ displacement due to respiration has been questioned [33, 37].

An experimental phantom was designed by Cleary et al. [10] to simulate the respiratory motion in liver. Bricault et al. [7] tracked respiratory motion using EM sensors integrated at the needles hub and investigated the relation between the needle position and the respiratory-induced motion of the target in the liver. The needle allowed the authors to predict the location of the moving targets. They also suggested that integrating the sensor into the needle tip instead of the hub is desirable to estimate the respiratory motion accurately. Borgert et al. [6] followed the findings of Bricault et al. and investigated another special needle with similar EM sensors on the needle tip and also on the patients sternum to estimate the target motion during a liver biopsy. The data acquired from EM sensors were used to evaluate the correlation between the positions of the two sensors and to derive a motion model to assess respiratory motion compensation for percutaneous needle interventions. Furthermore, Lei et al. [20] integrated multiple EM sensors along a flexible needle and used the movement and bending of the needle to estimate the liver motion during free and ventilator controlled breathing. However, a simplistic model was developed to correlate the needle motion/deformation to the target motion and the error was in the range of 2–4 mm [20].

The purpose of this study is to develop and evaluate an approach to estimate the position of a moving target caused by respiration that could improve targeting accuracy. For this purpose, we applied the concept of reconstruction of a correspondence model that creates a relationship between a respiratory surrogate signal and the target motions [15] to estimate the target position for percutaneous needle interventions. We also developed an experimental platform to mimic respiratory liver motion to experimentally validate the proposed approach. Our hypothesis is that the external motion of an initially placed guiding reference needle inserted into a moving organ can be used as a surrogate, measurable and accessible signal to estimate in real-time the position of a moving target caused by respiration [34]. The reference needle is the first needle inserted into the organ. Images of the reference needle are often used to guide the consequent needles toward the target. Since the relationship between the reference needle and the target is largely fixed throughout the respiratory cycle, it can be used to direct the subsequent needle insertions [29]. In this study, the concept of using the motion of the needle inserted into the moving organ as a surrogate signal was assessed using an inertial measurement unit (IMU) sensor attached to the hub of the reference needle.

**Fig. 1:**
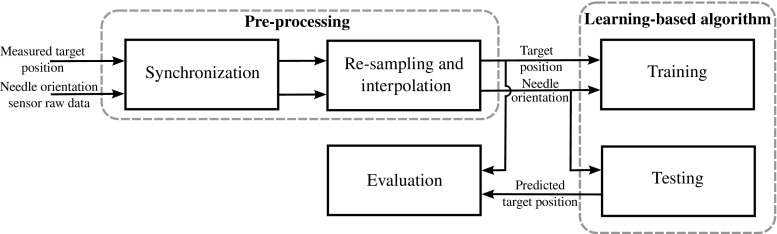
The needle position was measured for several respiratory cycles while measuring independently the target motion. The measured needle position (sensor) and target position (electromagnetic (EM) tracking) were preprocessed before being used for training the machine learning algorithm. The learning-based algorithm was then tested to estimate the target motion using only the needle position as an input. The target position obtained from the EM sensor was used as the gold standard to evaluate the output of the learning-based algorithm

**Fig. 2:**
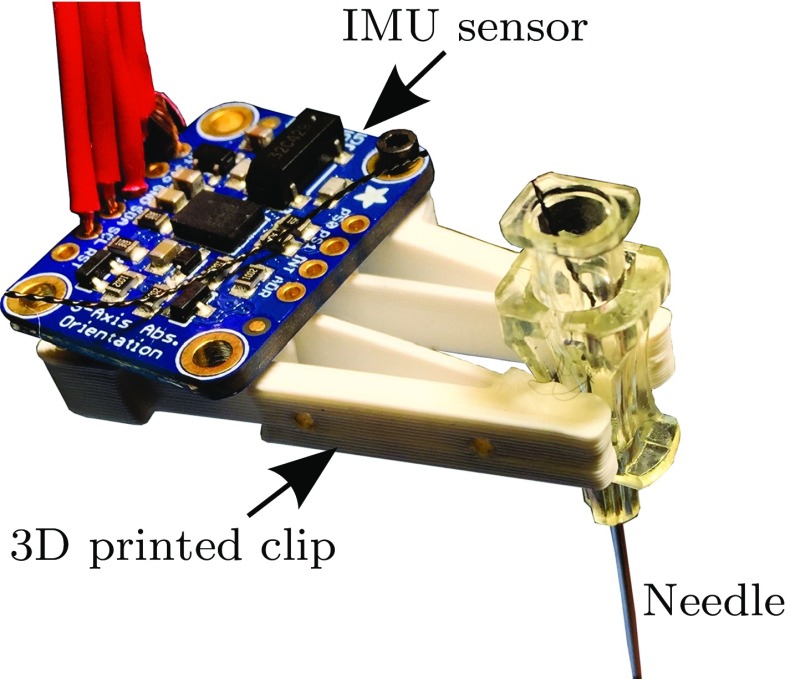
The inertial measurement unit (IMU) was attached to the biopsy needle inserted into the moving phantom

## Materials and methods

The aim of study was to assess our motion estimation approach using the surrogate signal obtained from reference needle and correspondence model generated by supervised machine learning. The study was conducted by constructing a special reference needed equipped with IMU at its hub and performing a mock procedure using moving phantom. We then varied respiratory motion profiles and conditions of needle placement to investigate their impact on the accuracy of target estimation. The estimation error was measured as difference between estimated location of the target by IMU and the correspondence model, and actual target location directly measured by an electromagnetic sensor (see Fig. 1).

### Reference needle as surrogate signal

Surrogate signal was collected from IMU (BNO055, Bosch Sensortec GmbH, Reutlingen, Germany) attached to the hub of the reference needle (see Fig. 2). The IMU has a triaxial 16-bit gyroscope, triaxial 14-bit accelerometer and a geomagnetic sensor. The signal output of the IMU was position, orientation, linear acceleration and angular velocity around its pitch, roll and yaw axes emitted at the sampling rate 100 Hz. A microcontroller board was used to obtain the signal from IMU (Arduino MICRO, Arduino, Italy) via an Inter-Integrated Circuit module. Serial communication was then used to connect the microcontroller board to the computer in order to record the measured data. The output signal from the IMU was used as an input to the correspondence model to estimate needle respiratory-induced motion of targets in liver from the surrogate signal.

### Target location as gold standard in training

The target position was measured using an electromagnetic (EM) sensor (Northern Digital Inc., Waterloo, Canada) at the target site. Another 5 degrees-of-freedom (DoF) EM sensor was also embedded into the reference needle inserted in the phantom to measure the distance between the reference needle tip and target, and also to measure the needle insertion angle. The target location, the distance between the needle tip and target, and the needle insertion angle were displayed and calculated using a free open-source medical image computing software, 3D Slicer [13]. The EM sensor data and the IMU sensor measurements were synchronized by applying a mechanical trigger that is detectable by both sensors. The data were then used as input to the learning-based algorithm (correspondence model) as described in the next subsection.

### Correspondence model trained by machine learning algorithm

The correspondence model attempts to model the relationship between the location of the target and the surrogate signal, which is the needle position, measured using the IMU sensor [25]. This can be written as,

1 $$\begin{aligned} {\mathbf {M(t)}=\phi ({\mathbf {s(t)}})} \end{aligned}$$

where ${\mathbf {s(t)}}$ is the surrogate data measured using the IMU sensor, $\phi$ the direct correspondence model (developed using the learning-based algorithm) and ${\mathbf {M(t)}}$ the estimate of the motion (a vector of the 3D target position at a certain time instance). The number of degrees of freedom of the model is determined by the number and nature of the surrogate data (${\mathbf {s}}$). The outputs of the IMU sensor are the position, orientation, linear acceleration and angular velocity around its pitch, roll and yaw axes. The surrogate values directly parameterise the motion/position estimates.

The corresponding model is established in the training phase where both surrogate signal and target location are measured as gold standard. The correspondence model established in this training phase is then applied to estimate the target motion during needle placement based only on newly obtained surrogate signal. In this particular study, the correspondence model was trained using a machine learning algorithm. Our machine learning algorithm is based on Random k-Labelsets (RAkEL) method for classification (multi-variant regression) [36]. The RAkEL method was selected as it showed higher performance compared to other popular multi-label classification methods such as Binary Relevance and label powersets methods [36]. *k* is a parameter that specifies the size of the labelsets. The main idea in this method is to randomly break a large set of labels into a number of small-sized labelsets, and for each of labelsets train a multi-label classifier using the label powerset method. Disjoint labelset construction version RAkELd presented in [35] was used in the current study as it can process multiple numeric inputs (surrogate data) and outputs (target position). For training and testing the correspondence model, we used the open-source MEKA software [28]. The multi-label learning-based classification software was used to generate the correspondence model and then estimate the target position in 3D space. The measured needle position and target motion at each instance during respiratory motion represents a single training point for the learning algorithm.

### Target estimation

The target position was estimated by supervised training of data. Three training methods were applied for target motion estimation. First, train/test split (TTS) methods of 20 s of the data were performed to evaluate the learning algorithm where 66% of the data points were used for training and 34% was used for testing. Training data of more than one respiratory cycle were selected to account for variations in different breathing patterns within the same subject. The data points used for training and testing the learning algorithm were selected randomly in order to consider the variation in the respiratory motion profile in the collected data. Second, cross-validation (CV) was also performed where a data set of 140 s was used for training and then complete data set was tested in the same order (without randomization). The aim of the CV testing is to estimate the processing time for large number of data points and also to test the correlation between the estimation error and the respiratory phase. Third, cross-validation with delay (CVD) was performed. Based on the rate by which the clinician will visually receive change in the target position, a delay of 20 ms is included to determine the effect of the delay on the accuracy of target estimation [12]. After training, the correspondence model used only the surrogate signal from the IMU sensor to estimate the 3-dimensional (3D) position of the target at a certain moment during respiration. The actual target position obtained from the EM sensors (embedded into the phantom) was only used for measuring the estimation error.

### Phantom

**Fig. 3:**
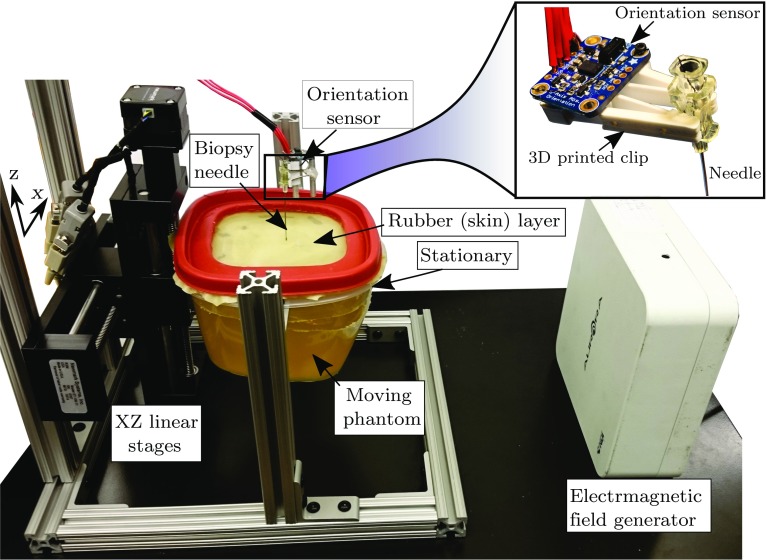
Experimental setup: the inertial measurement unit (IMU) was attached to the biopsy needle inserted into the moving phantom. Electromagnetic tracking sensor was embedded into the gelatin phantom to measure the target motion. The phantom motion (11 cm height) was actuated using 2D motorized linear stages. The motion of the upper part of the phantom that includes a rubber layer (skin) was constraint

A gelatin-based phantom was used mimic the elasticity of human liver. The gelatin-to-water mixture (1.6 L) of 15% (by weight) was used (Knox gelatin, Kraft Foods Group, Inc, Illinois, USA). The phantom was placed in a container and covered by an abrasion-resistant natural latex rubber layer (McMaster-Carr, Illinois, USA) of 0.5 mm thickness to mimic the skin. To simulate the respiratory motion, the skin layer and the upper part (2 cm) of the gelatin phantom were clamped in the *x*- and *y*-directions but can move in the *z*-direction (up and down). The phantom was attached to two motorized stages (type eTrack ET-100-11, Newmark Systems Group Inc. California, USA) actuated with stepper motors to simulate the respiratory motion in liver. Both motors were controlled using a Newmark controller NSC-A2L Series (see Fig. 3).

**Fig. 4:**
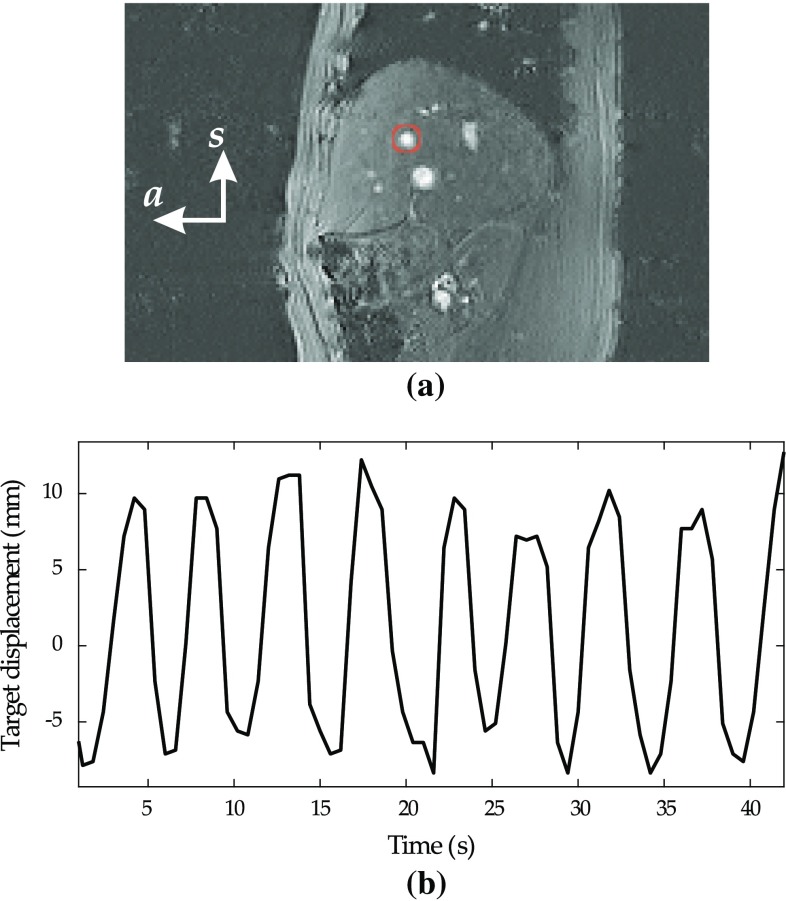
**a** Sample image of the liver with a highlighted target. The anterior (*a*) and superior (*s*) axes are presented in the figure. **b** A plot of the target motion during respiration in the vertical direction

### Motion profile

The target motion range and the velocity were selected based on the results obtained from the MR images of eight human subjects recruited. Informed consent was obtained according to an IRB-approved protocol and performed in accordance with ethical standards. Sagittal images were obtained using a steady-state gradient-echo sequence from the human subjects scanned in 3T wide-bore MRI scanner (MAGNETOM Verio 3T, Siemens, Erlangen, Germany) to measure the liver respiratory motion (see Fig. 4). In the liver MR images, the motion of three structures (blood vessels) that resembled the lesion was tracked in each MR image frame. These structures were located manually in each image frame to determine the target motion during respiration. The image slice thickness flip angle matrix size and field of view were 5 mm, 30$^\circ$, $192 \times 192$ and $38 \times 38$ cm$^2$, respectively. The frequency of acquiring images was 1 Hz, and the duration of each scan was $140.27 \pm 51.10$ s. Per scan, $122 \pm 45.86$ images were acquired. The tracked target motion in MR images shows that the motion was mainly in the anterior-posterior and inferior-superior axis; $8.10 \pm 4.71$ and $2.53 \pm 1.60$ mm, respectively (see Fig. 4). The mean velocities of target motion were $3.54 \pm 1.044$ and $1.11 \pm 0.33$ mm/s in the anterior-posterior and inferior-superior axis, respectively.

The anterior–posterior and inferior–superior motions (displacement and velocity) obtained from the subjects’ MRI data were set to the controllers of the two motorized linear stages of the phantom. Target motion varies among subjects and also at respiratory cycles of same subject. A random fraction was added to the mean motion value to account for variation of motion during simulations.

### Data collection

Experiments were performed to determine the accuracy of the developed motion estimation approach at different insertion angles, target depths, target motion velocities and target proximity to the needle. The initial parameters are: 60$^\circ$ insertion angle, 8 cm target depth, 3.5 mm/s target velocity, 1–2 cm distance between the needle tip and target. The experimental conditions are presented in Table 1. The target displacement was measured using the EM-tracker, and needle motion was measured using the IMU sensor. The steps of data synchronization and processing are presented in Fig. 1. Each experiment was performed seven times. The results were evaluated by calculating the error between the estimated error from the learning-based algorithm and the gold standard, which was the actual target position, obtained from the EM sensor.

**Table 1:** Experimental protocol for validation of the correspondence model while varying the motion profiles and the conditions are presented

Experiment testing the effect of	Insertion angle ($^{\circ }$)			Target depth (cm)			Target velocity (mm/s)			Proximity to needle (cm)		
	40	60	90	4	8	10	2.5	3.5	4.5	0–1	1–2	2–3
Insertion angle	$\checkmark$	$\checkmark$	$\checkmark$		$\checkmark$			$\checkmark$			$\checkmark$	
Target depth		$\checkmark$		$\checkmark$	$\checkmark$	$\checkmark$		$\checkmark$			$\checkmark$	
Target velocity		$\checkmark$			$\checkmark$		$\checkmark$	$\checkmark$	$\checkmark$		$\checkmark$	
Proximity to needle		$\checkmark$			$\checkmark$			$\checkmark$		$\checkmark$	$\checkmark$	$\checkmark$

### Processing time

The processing time of the training and testing the learning-based algorithm was measured to determine expected delay of the motion estimation algorithm. Kruskal–Wallis test was performed to determine the statistical significance of the tested parameters as it can deal with more than two data sets [18].

## Results

**Fig. 5:**
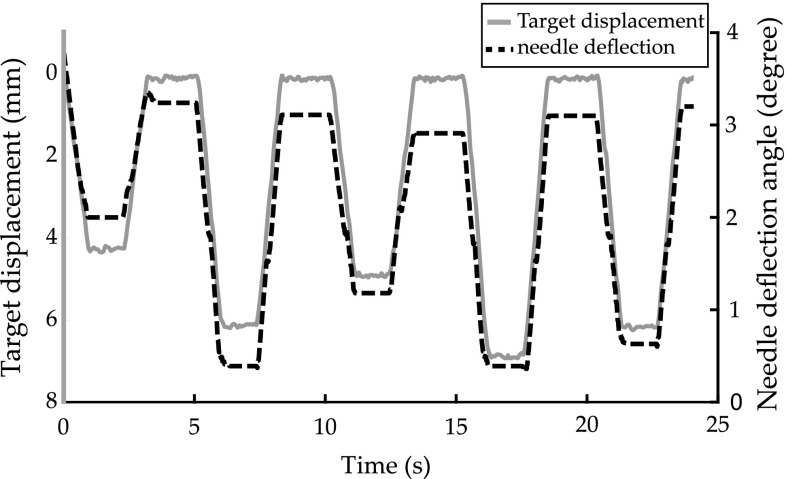
Sample data of the needle deflection and the absolute displacement of the target during respiratory motion

This section presents the experimental results of the validation study to evaluate the target motion estimated using the correspondence model. The results obtained from the learning algorithm are presented in Table 2. Figure 5 shows the target motion with respect to the needle deflection (raw surrogate signal) during respiratory motion. The estimation error was measured as the absolute distance between the estimated position of the target (obtained from the learning-based algorithm) and the actual position of the target (measured using EM trackers embedded at the target site). The training time of the learning-based algorithm was in the range of 4 ms, while the testing time was 1 ms. The motion estimation error while including the delay affected the error by a magnitude of less than 0.01 mm which is negligible. The insignificant effect of the delay is due to the relatively slow pace of respiration with respect to quick response of the interventional radiologist to a visual update of the target position.

**Table 2:** Experimental results for validation of the correspondence model while varying the motion profiles and the conditions are presented

Evaluation method	Insertion angle ($^{\circ }$)			Target depth (cm)			Target velocity (mm/s)			Proximity to needle (cm)		
	40	60	90	4	8	10	2.5	3.5	4.5	0–1	1–2	2–3
TTS	$1.08\pm 0.48$	$1.04\pm 0.46$	$0.86\pm 0.53$	$1.08\pm 0.43$	$1.04\pm 0.46$	$1.08\pm 0.46$	$1.29\pm 0.37$	$1.04\pm 0.46$	$1.21\pm 0.41$	$1.10\pm 0.49$	$1.04\pm 0.46$	$0.90\pm 0.44$
CV	$1.24\pm 0.51$	$1.12\pm 0.53$	$0.94\pm 0.56$	$1.15\pm 0.44$	$1.12\pm 0.53$	$1.10\pm 0.43$	$1.16\pm 0.45$	$1.12\pm 0.53$	$0.86\pm 0.45$	$1.30\pm 0.37$	$1.12\pm 0.53$	$1.23\pm 0.41$
CVD	$1.25\pm 0.51$	$1.12\pm 0.53$	$0.94\pm 0.56$	$1.15\pm 0.44$	$1.12\pm 0.53$	$1.11\pm 0.42$	$1.17\pm 0.45$	$1.12\pm 0.53$	$0.87\pm 0.45$	$1.30\pm 0.37$	$1.12\pm 0.53$	$1.23\pm 0.41$

The estimation error is presented in mm, and it is the absolute distance between the actual target position and its estimated position at a certain moment during respiration. The evaluation methods for evaluating the estimated error are train/test split (TTS), cross-validation (CV) and also using CV with a delay (CVD). Each experiment was repeated seven times

**Fig. 6:**
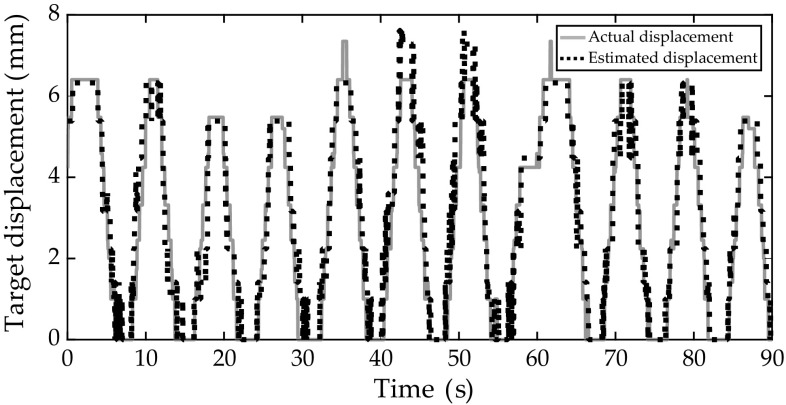
Sample plot that shows the actual target displacement acquired from the electromagnetic sensor and the estimated target displacement from the correspondence model

The results show that the target estimation error varied within a limited range and the mean errors of the experimental trials varied between 0.86 mm and 1.29 mm. It was observed that applying; (1) needle insertion angles of 40$^\circ$, 60$^\circ$ and 90$^\circ$ ($p<0.001$), (2) target depths of 4, 8 and 10 cm ($p<0.001$) and, (3) target-to-needle tip distance of 0–1, 1–2 and 2–3 cm did not show significant change in the estimated a targeting error. We could not also conclude from the results that the target velocity does significantly affect absolute target error ($p=0.021$). Figure 6 shows the actual and estimated target position during respiration. It can be observed from the results that the error was more significant at extreme inhalation.

## Discussion

In this study we developed and evaluated a novel approach to estimate the respiratory motion of lesions in the chest an upper abdomen during percutaneous needle intervention. With the liver as a model, we used the motion of an initially placed reference needle as a surrogate signal and used machine learning as a correspondence model to estimate the position of a target during respiration. The motion of the reference needle was measured using an IMU sensor attached to the needle hub. To validate the proposed approach, an experimental platform was designed to simulate the liver motion based on MRI data collected from human subjects. A number of variables including the insertion angle, target depth, target velocity and target proximity to the needle were used to evaluate the correspondence model. The experimental results showed that the mean error of estimation of the target position ranges between 0.86–1.29 mm. The maximum processing time for training is 4 ms, and for testing is 1 ms which is suitable for real-time target motion estimation using the IMU sensor attached to the needle. From our experimental observations, we found that at low insertion depths, the sensor data were more sensitive to factors other than the target motion such as the weight of sensors and cables attached to the needle as the needle was not inserted deep enough to be fixed in the moving organ. The proposed approach can be applicable in a clinical work flow by using the IMU sensor to estimate the motion. However for MRI-guided needle interventions, an MRI-compatible needle should be used. Additionally, MRI-compatible IMU sensors can be used such as the sensor that was recently presented by Chen et al. [9] where optical fibers were used for communication to prevent the introduction of MRI image artifacts. In the first phase, a sequence of MRI images will be needed for few respiratory cycles to develop the correspondence model using supervised learning (training the learning-based algorithm). In the next phase, only IMU data will be used as an input to the trained model to estimate target motion in real-time. The target motion will be provided to the interventional radiologist to compensate for this motion and thus steer the needle accurately toward the target.

Further improvements are needed to enhance the proposed approach to minimize the estimation error. One of the limitations of the presented approach is that it is sensitive to needle bending that can occur in the moving tissue. The needle bending in biological tissue can affect direct relation between the external motion of the needle hub and the actual motion of the needle tip (close to the target). Additionally, a more specific model can be used that includes the various layers of tissue the needle penetrates till it reached the liver capsule. This model should consider the elastic and mechanical properties of each layer and also its motion constraints. The proposed approach was validated while considering shallow breathing. This needs to be extended to include a variety of breathing patterns and also propose methods to feedback or display the target motion to the clinician to account for the motion during the interventional procedure. To take the proposed approach a step toward clinical practice, the proposed approach should be validated in non-homogeneous tissue (biological tissue) and also in animal studies.
